# The application of magnetic nanoparticles for the treatment of brain tumors

**DOI:** 10.3389/fchem.2014.00109

**Published:** 2014-12-03

**Authors:** Keon Mahmoudi, Costas G. Hadjipanayis

**Affiliations:** ^1^Georgia Institute of Technology, School of BiologyAtlanta, GA, USA; ^2^Brain Tumor Nanotechnology Laboratory, Department of Neurosurgery, Winship Cancer Institute of Emory University, Emory University School of MedicineAtlanta, GA, USA

**Keywords:** nanotheranostics, magnetic nanoparticles, hyperthermia, nanoparticles, imaging, glioblastoma

## Introduction

Glioblastoma (GBM), a World Health Organization (WHO) grade IV astrocytoma, is the most common and difficult primary brain tumor to treat (Braun et al., 2012). Even when detected early, the median survival rate for patients is 12–15 months (Adamson et al., 2009; Johnson and O'Neill, 2012). The challenge in treating GBM arises from its resistance to therapies such as radiotherapy and chemotherapy. GBM tumors are quite infiltrative into the surrounding normal brain permitting tumors to recur locally in the majority of patients.

The current standard of care treatment for GBM involves surgery and radiation, with concurrent and adjuvant chemotherapy (Stupp et al., 2005). Surgery permits the bulk of a GBM tumor to be removed in most cases. All patients have residual tumor cells residing away from the resection cavity that eventually lead to local tumor recurrence and the demise of the majority of patients (Hou et al., 2006). The infiltrating GBM cells reside centimeters away from the main tumor mass in normal brain making it difficult for complete surgical removal (Kim et al., 2014). Chemotherapy and radiotherapy of patients after surgery attempts to target these cells to prolong overall patient survival. The blood brain barrier (BBB) represents another challenge to the treatment of GBM tumors by preventing the accumulation of most chemotherapeutics into the brain to target the infiltrative cancer cells (Salazar et al., 1976; Bidros and Vogelbaum, 2009). Surgery and adjuvant therapies pose risks to the patient such as neurologic deficits and systemic toxicities. Known side effects of radiation therapy with chemotherapy for brain tumors include chronic fatigue, nausea, and cognitive deficits (Loehrer et al., 2011).

The BBB remains a formidable challenge in the treatment of GBM and malignant brain tumors. Its selective permeability is due to the presence of specialized endothelial cells, astrocytes, pericytes, and neuronal terminals (Tajes et al., 2014). The semi-permeable membrane that comprises the BBB prevents sufficient exposure of tumors to most chemotherapeutic drugs that are commonly used to fight tumor progression (Liu et al., 2010). Local disruption of the BBB is found within GBM tumors. The tumor vessels in GBM tumors are abnormal both structurally and functionally (Batchelor et al., 2007). The abnormal tumor vessels further impair delivery of therapeutics and create a hypoxic microenvironment that can reduce the effectiveness of radiation and chemotherapy. Antiangiogenic therapy attempts to normalize the tumor vasculature and improve the tumor microenvironment (Jain, 2001, 2005). Outside of the main tumor mass, the BBB is intact where brain cancer cells infiltrate into the surrounding normal brain. The oral chemotherapy agent, temozolomide (Temodar), can penetrate the BBB and has resulted in prolongation of overall survival patient survival by several months (Stupp et al., 2005).

The challenges associated with the treatment of GBM tumors require novel approaches for a greater impact on patient survival and quality of life for patients.

## Magnetic nanoparticles (MNPs)

MNPs are most commonly comprised of ferromagnetic iron-oxide (Fe_3_O_4_). They are invisible to the naked eye, typically measuring 1–100 nm in diameter (Sandhiya et al., 2009). MNPs can be designed to target cancer by modification of their surface with the addition of a peptide or antibody specific to cancer cells (Hadjipanayis et al., 2010). For biomedical applications, they can deliver targeted therapy to specific regions of the body. MNPs can be administered into the blood stream systemically and directed to a target with application of an external magnetic field (Pankhurst et al., 2003). Particles can be engineered to carry a drug, which can be released once the particles reach their target. *In vivo* experiments have shown the effects of MNPs within a magnetic field on glioma cells lasting up to 100 min postexposure (Braun et al., 2012). In a separate study with rabbits, intravenous injection of specially designed MNPs and subsequent exposure to an external magnetic field resulted in permanent remission of squamous cell carcinoma tumors (Chertok et al., 2008). While intravenous administration is feasible with tumors in other parts of the body, the BBB remains a formidable challenge for systemic delivery of agents for treatment of brain tumors. For the treatment of patients with GBMs, direct intratumoral delivery provides the greatest concentration of therapeutic while minimizing systemic toxicities.

### MRI contrast enhancement of brain tumors

MNPs also serve as a powerful aid for the imaging of brain tumors. Their inherent ferromagnetic qualities provide sensitive contrast enhancement with MR imaging (Liu et al., 2010). Accumulation of MNPs in brain tumors appears as a hypointensity on T2-weighted imaging including gradient echo imaging (Na et al., 2007). Functionalized MNPs can be engineered to target brain cancer cells which can in turn be identified with MR imaging. For standard visualization of tumors, MNPs can provide more sensitive imaging of tumors when used as a contrast agent for MRI (Kumar et al., 2010).

Ultrasmall superparamagnetic iron oxide nanoparticles (USPIONPs), a subclass of superparamagnetic MNPs, are the most effective types of MNPs that can be used for imaging purposes (Thorek et al., 2006). Their systemic half-life is two to three times greater than standard MNPs and are capable of being imaged by MRI for longer periods of time (Varallyay et al., 2002). In a recent study, it was noted that USPIONPs can be used to detect areas within brain tumors with increased blood flow, which may be indicative of tumor recurrence (Gambarota and Leenders, 2011). They can also be used to identify areas of pseudoprogression in brain tumors after standard adjuvant therapies such as radiotherapy and chemotherapy (Gahramanov et al., 2011).

## Hyperthermia

Hyperthermia for the treatment of different cancers has been well described in the past. Elevation of targeted areas of the body above 40°C can result in cancer cell death (Wust et al., 2002). In one study, researchers concluded that even moderate hyperthermia at a temperature around 45°C was enough to cause tumor cells to undergo apoptosis (Pu et al., 2013). Furthermore, local or regional hyperthermia can result in elevated blood flow, which may assist in the delivery of other treatments, such as chemotherapy, which could result in a synergistic antitumor effect (Kampinga, 2006; Issels, 2008).

While local or regional hyperthermia can be effective in treating cancer involving different parts of the body, treating brain tumors is difficult due to the surrounding skull (Jordan et al., 1999). Heat applied to the head is shielded by the skull which results in less than optimal temperature increases in the brain. Temperature elevation of the entire brain for prolonged periods of time would result in side effects and toxicities to patients. To provide a more targeted hyperthermia effect for brain tumors, MNPs may be delivered intratumorally prior to treatment with alternating magnetic fields. This process, known as thermotherapy, aims to deliver a greater hyperthermia effect locally to brain tumors while minimizing heating of the surrounding brain.

### Thermotherapy

**Figure 1:**
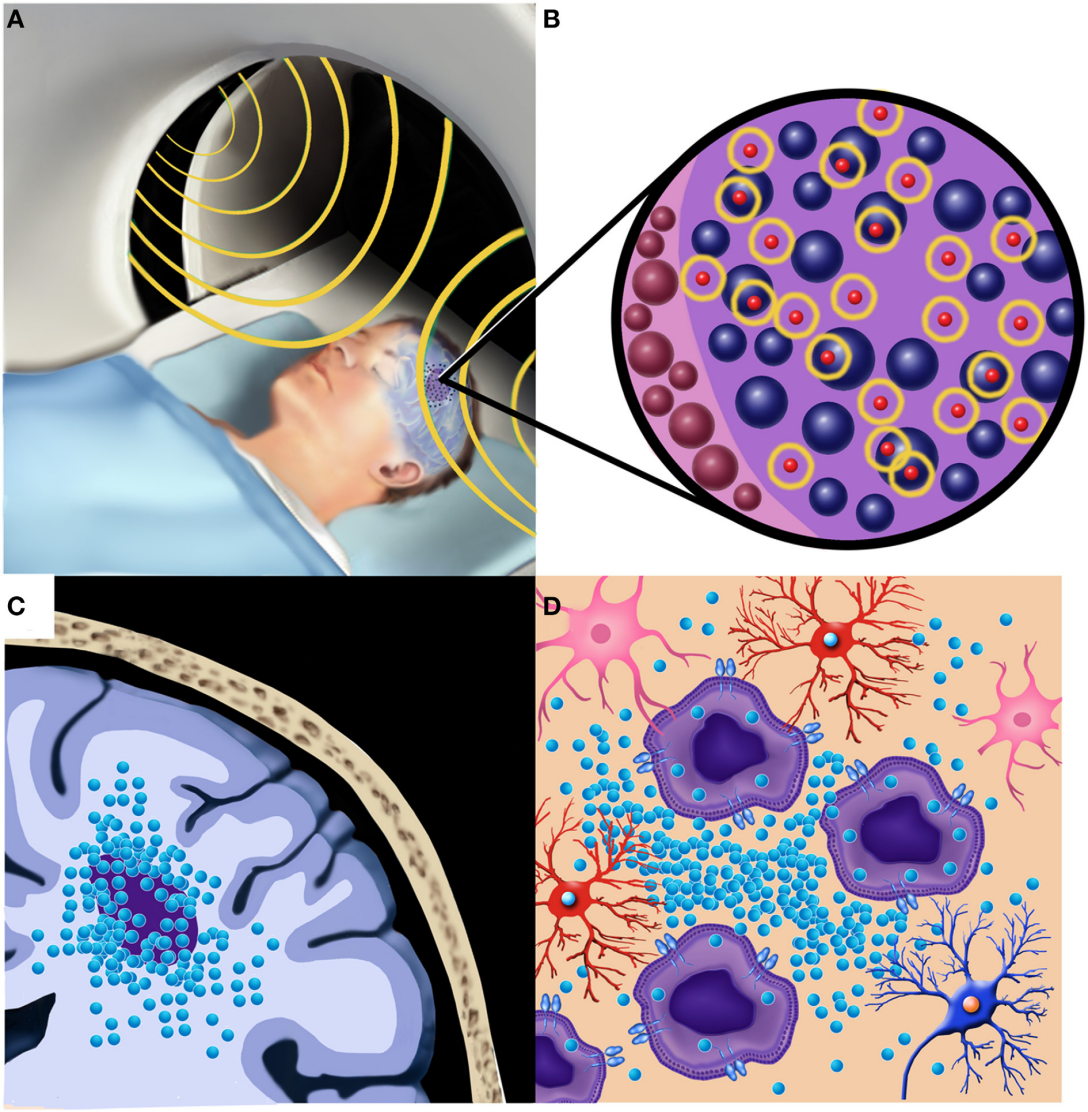
**Local hyperthermia treatment of a patient with a malignant brain tumor after implantation of MNPs**. **(A)** The patient undergoes an alternating magnetic field (AMF) session (shown in yellow) for generation of local hyperthermia in the region of the tumor. **(B)** Oscillation of the MNPs (shown in yellow) within and adjacent to the tumor cells provides the therapeutic hyperthermia (thermotherapy) effect. **(C)** Local implantation of the MNPs within and adjacent to the brain tumor provides a targeted therapeutic effect. **(D)** Brain tumor cells shown infiltrating normal brain may be more susceptible to the effects of local hyperthermia by greater MNP intracellular uptake and sensitivity to temperature changes.

Due to the side effects and toxicities of subjecting the entire brain to hyperthermia for extended periods of time, localized treatment is necessary for effective brain tumor therapy. Direct implantation of MNPs into brain tumors can bypass the BBB and allow for a maximum hyperthermic effect provided in a targeted manner Figure 1. Brain autopsies of two GBM patients after MNP injection confirmed that the MNPs were retained within tumor tissue after implantation (Van Landeghem et al., 2009). Once injected into tumors, MNPs are subjected to an alternating magnetic field (AMF) which produces heat via the Brownian Néel relaxation process (Thiesen and Jordan, 2008; Deissler et al., 2014).

The localized hyperthermic effect, known as thermotherapy, involves the application of an alternating magnetic field (Maier-Hauff et al., 2011) Figure 1. When applying a magnetic field to the target area, the strength of the hyperthermic treatment is dependent on a variety of factors including the strength of the AMF, the size and concentration of the MNPs, and the time in which the field is applied to the tumor region (Yanase et al., 1997; Guedes et al., 2004; Meenach et al., 2010). Targeted treatment is necessary because prolonged application of hyperthermia to healthy tissue can result in unwanted side effects and toxicities (Fajardo, 1984). In order to minimize the risk of systemic toxicities, the hyperthermic treatment is only applied for a brief period of time to allow for the MNPs within the targeted region to heat up and cause necrosis or death of the cancer cells. In human patients with brain tumors, it was determined that hyperthermia with temperatures from 42°C to 49°C were safe and caused very few side effects for the patient (Maier-Hauff et al., 2007).

Thermotherapy does induce the death of malignant cells (Marcos-Campos et al., 2011). When a MNP is subjected to an alternating magnetic field, its internal temperature increases. This heat is then transferred locally to the abnormal cells situated around the nanoparticles which further results in tumor death (Fajardo, 1984). With thermotherapy, only the targeted tumor region is exposed to increased temperatures, resulting in localized necrosis. When clinicians studied the benefits of using thermotherapy in conjunction with radiotherapy in relapsed GBM, they reported an overall survival of 13.4 months compared to just 6.2 months with radiotherapy and Temozolomide alone (Maier-Hauff et al., 2011). Current limitations to the use of MNPs for thermotherapy of brain tumor patients include the high MNP concentration required to generate hyperthermia precluding the use of MRI, as well as the effective delivery of the MNPs (Wankhede et al., 2012).

The decreased resistance to heat observed in GBM cells is not as clearly presented when conducting experiments with *in vitro* samples (Issels, 2008). Cancer cells that reside in tumors are more susceptible to damage from heat than cancer cells that are *in vitro* (Rhee et al., 1990). This contrasts heavily with the significant difference in immunity that is observed when experiments are conducted using *in vivo* models. One explanation for this difference is that the vascular network within the tumor is abnormal which can lead to areas that have a difference in pH as well as decreased availability of oxygen (Issels, 2008).

## Conclusion

Thermotherapy involving the use of an AMF in conjunction with MNPs has proven to be an effective method for treating patients with GBM. Initial tests have shown that MNPs have minimal toxicities to patients, though further testing must be done to confirm these findings (Mahmoudi et al., 2012). Much like other methods that are used to combat GBM, MNPs do not serve as a cure on their own; they have shown to be most effective when used as an adjuvant therapy with other treatment modalities. Combining fractionated radiotherapy with thermotherapy has shown a survival advantage in patients with relapsed GBM (Maier-Hauff et al., 2011).

With further research, scientists can bioengineer multitasking MNPs that can be used for imaging, drug delivery, and localized thermotherapy (Hadjipanayis et al., 2013). Better targeting of MNPs may provide more effective treatment of GBM. The bioconjugation of drugs, monoclonal antibodies, or peptides specific to cancer cells will improve targeting. MNPs appear to be well tolerated when delivered directly into the human brain with few side effects associated with them. Further testing of MNPs with standard of care chemotherapy, such as temozolomide, needs to be completed in patients with malignant brain tumors. MNPs will likely assume a larger role in brain cancer treatment, with other adjuvant therapies being used to complement magnetic nanoparticles (Kim et al., 2014).

### Conflict of interest statement

The authors declare that the research was conducted in the absence of any commercial or financial relationships that could be construed as a potential conflict of interest.

## References

[B1] Adamson C. Kanu O. O. Mehta A. I. Di C. Lin N. Mattox A. K.. (2009). Glioblastoma multiforme: a review of where we have been and where we are going. Expert Opin. Investig. Drugs 18, 1061–1083. 10.1517/13543780903052764 19555299

[B2] Batchelor T. T. Sorensen A. G. Di Tomaso E. Zhang W. T. Duda D. G. Cohen K. S.. (2007). AZD2171, a pan-VEGF receptor tyrosine kinase inhibitor, normalizes tumor vasculature and alleviates edema in glioblastoma patients. Cancer Cell 11, 83–95. 10.1016/j.ccr.2006.11.021 17222792 PMC2748664

[B3] Bidros D. S. Vogelbaum M. A. (2009). Novel drug delivery strategies in neuro-oncology. Neurotherapeutics 6, 539–546. 10.1016/j.nurt.2009.04.004 19560743 PMC5084189

[B4] Braun S. Oppermann H. Mueller A. Renner C. Hovhannisyan A. Baran-Schmidt R.. (2012). Hedgehog signaling in glioblastoma multiforme. Cancer Biol. Ther. 13, 487–495. 10.4161/cbt.19591 22406999

[B5] Chertok B. Moffat B. A. David A. E. Yu F. Bergemann C. Ross B. D.. (2008). Iron oxide nanoparticles as a drug delivery vehicle for MRI monitored magnetic targeting of brain tumors. Biomaterials 29, 487–496. 10.1016/j.biomaterials.2007.08.050 17964647 PMC2761681

[B6] Deissler R. J. Wu Y. Martens M. A. (2014). Dependence of brownian and neel relaxation times on magnetic field strength. Med. Phys. 41, 012301. 10.1118/1.4837216 24387522

[B7] Fajardo L. F. (1984). Pathological effects of hyperthermia in normal tissues. Cancer Res. 44, 4826s–4835s. 6467235

[B8] Gahramanov S. Raslan A. M. Muldoon L. L. Hamilton B. E. Rooney W. D. Varallyay C. G.. (2011). Potential for differentiation of pseudoprogression from true tumor progression with dynamic susceptibility-weighted contrast-enhanced magnetic resonance imaging using ferumoxytol vs. gadoteridol: a pilot study. Int. J. Radiat. Oncol. Biol. Phys. 79, 514–523. 10.1016/j.ijrobp.2009.10.072 20395065 PMC3111452

[B9] Gambarota G. Leenders W. (2011). Characterization of tumor vasculature in mouse brain by USPIO contrast-enhanced MRI. Methods Mol. Biol. 771, 477–487. 10.1007/978-1-61779-219-9_25 21874494

[B10] Guedes M. H. A. Guedes M. E. A. Morais P. C. Da Silva M. F. Santos T. S. Alves J. P. Jr. (2004). Proposal of a magnetohyperthermia system: preliminary biological tests. J. Magn. Magn. Mat. 272-276(Pt 3), 2406–2407 10.1016/j.jmmm.2003.12.709

[B11] Hadjipanayis C. G. Bouras A. Chang S. (2013). Applications of multifunctional nanoparticles in malignant brain tumours. Eur. Assoc. Neurooncol. Mag. 4, 9–15.

[B11a] Hadjipanayis C. G. Machaidze R. Kaluzova M. Wang L. Schuette A. J. Chen H.. (2010). EGFRvIII antibody-conjugated iron oxide nanoparticles for magnetic resonance imaging-guided convection-enhanced delivery and targeted therapy of glioblastoma. Cancer Res. 70, 6303–6312. 10.1158/0008-5472 20647323 PMC2912981

[B12] Hou L. C. Veeravagu A. Hsu A. R. Tse V. C. (2006). Recurrent glioblastoma multiforme: a review of natural history and management options. Neurosurg. Focus 20, E5. 10.3171/foc.2006.20.4.2 16709036

[B13] Issels R. D. (2008). Hyperthermia adds to chemotherapy. Eur. J. Cancer 44, 2546–2554. 10.1016/j.ejca.2008.07.038 18789678

[B14] Jain R. K. (2001). Normalizing tumor vasculature with anti-angiogenic therapy: a new paradigm for combination therapy. Nat. Med. 7, 987–989. 10.1038/nm0901-987 11533692

[B15] Jain R. K. (2005). Normalization of tumor vasculature: an emerging concept in antiangiogenic therapy. Science 307, 58–62. 10.1126/science.1104819 15637262

[B16] Johnson D. O'Neill B. (2012). Glioblastoma survival in the United States before and during the temozolomide era. J. Neuro Oncol. 107, 359–364. 10.1007/s11060-011-0749-4 22045118

[B17] Jordan A. Scholz R. Wust P. Fähling H. Roland F. (1999). Magnetic fluid hyperthermia (MFH): cancer treatment with AC magnetic field induced excitation of biocompatible superparamagnetic nanoparticles. J. Magn. Magn. Mat. 201, 413–419. 10.1016/S0304-8853(99)00088-815726645

[B18] Kampinga H. H. (2006). Cell biological effects of hyperthermia alone or combined with radiation or drugs: a short introduction to newcomers in the field. Int. J. Hyperthermia 22, 191–196 10.1080/02656730500532028 16754338

[B19] Kim S.-S. Rait A. Kim E. Pirollo K. F. Nishida M. Farkas N.. (2014). A nanoparticle carrying the p53 gene targets tumors including cancer stem cells, sensitizes glioblastoma to chemotherapy and improves survival. ACS Nano 8, 5494–5514. 10.1021/nn5014484 24811110 PMC4076028

[B20] Kumar M. Medarova Z. Pantazopoulos P. Dai G. Moore A. (2010). Novel membrane-permeable contrast agent for brain tumor detection by MRI. Magn. Reson. Med. 63, 617–624. 10.1002/mrm.22216 20146231 PMC3819103

[B21] Liu H.-L. Hua M.-Y. Yang H.-W. Huang C.-Y. Chu P.-C. Wu J.-S.. (2010). Magnetic resonance monitoring of focused ultrasound/magnetic nanoparticle targeting delivery of therapeutic agents to the brain. Proc. Natl. Acad. Sci. U.S.A. 107, 15205–15210. 10.1073/pnas.1003388107 20696897 PMC2930577

[B22] Loehrer P. J. Sr. Feng Y. Cardenes H. Wagner L. Brell J. M. Cella D.. (2011). Gemcitabine alone versus gemcitabine plus radiotherapy in patients with locally advanced pancreatic cancer: an eastern cooperative oncology group trial. J. Clin. Oncol. 29, 4105–4112. 10.1200/JCO.2011.34.8904 21969502 PMC3525836

[B23] Mahmoudi M. Hofmann H. Rothen-Rutishauser B. Petri-Fink A. (2012). Assessing the *in vitro* and *in vivo* toxicity of superparamagnetic iron oxide nanoparticles. Chem. Rev. 112, 2323–2338. 10.1021/cr2002596 22216932

[B24] Maier-Hauff K. Rothe R. Scholz R. Gneveckow U. Wust P. Thiesen B.. (2007). Intracranial thermotherapy using magnetic nanoparticles combined with external beam radiotherapy: results of a feasibility study on patients with glioblastoma multiforme. J. Neurooncol. 81, 53–60. 10.1007/s11060-006-9195-0 16773216

[B25] Maier-Hauff K. Ulrich F. Nestler D. Niehoff H. Wust P. Thiesen B.. (2011). Efficacy and safety of intratumoral thermotherapy using magnetic iron-oxide nanoparticles combined with external beam radiotherapy on patients with recurrent glioblastoma multiforme. J. Neurooncol. 103, 317–324. 10.1007/s11060-010-0389-0 20845061 PMC3097345

[B26] Marcos-Campos I. Asín L. Torres T. E. Marquina C. Tres A. Ibarra M. R.. (2011). Cell death induced by the application of alternating magnetic fields to nanoparticle-loaded dendritic cells. Nanotechnology 22:205101. 10.1088/0957-4484/22/20/205101 21444956

[B27] Meenach S. A. Hilt J. Z. Anderson K. W. (2010). Poly(ethylene glycol)-based magnetic hydrogel nanocomposites for hyperthermia cancer therapy. Acta Biomater. 6, 1039–1046. 10.1016/j.actbio.2009.10.017 19840875

[B28] Na H. B. Lee J. H. An K. Park Y. I. Park M. Lee I. S.. (2007). Development of a T1 contrast agent for magnetic resonance imaging using MnO nanoparticles. Angew. Chem. Int. Ed. Engl. 46, 5397–5401. 10.1002/anie.200604775 17357103

[B29] Pankhurst Q. A. Connolly J. Jones S. K. Dobson J. (2003). Applications of magnetic nanoparticles in biomedicine. J. Phys. D 36, R167 10.1088/0022-3727/36/13/201

[B30] Pu P.-Y. Zhang Y.-Z. Jiang D.-H. (2013). Apoptosis induced by hyperthermia in human glioblastoma cell line and murine glioblastoma. Chin. J. Cancer Res. 12, 257–262 10.1007/BF02983501

[B31] Rhee J. G. Eddy H. A. Harrison G. H. Salazar O. M. (1990). Heat-sensitive state of mouse mammary carcinoma cells in tumors. Radiat. Res. 123, 165–170. 10.2307/3577540 2389002

[B32] Salazar O. M. Rubin P. McDonald J. V. Feldstein M. L. (1976). High dose radiation therapy in the treatment of glioblastoma multiforme: a preliminary report. Int. J. Radiat. Oncol. Biol. Phys. 1, 717–727. 10.1016/0360-3016(76)90155-3 185174

[B33] Sandhiya S. Dkhar S. A. Surendiran A. (2009). Emerging trends of nanomedicine–an overview. Fundam. Clin. Pharmacol. 23, 263–269. 10.1111/j.1472-8206.2009.00692.x 19527298

[B34] Stupp R. Mason W. P. Van Den Bent M. J. Weller M. Fisher B. Taphoorn M. J.. (2005). Radiotherapy plus concomitant and adjuvant temozolomide for glioblastoma. N. Engl. J. Med. 352, 987–996. 10.1056/NEJMoa043330 15758009

[B35] Tajes M. Ramos-Fernandez E. Weng-Jiang X. Bosch-Morato M. Guivernau B. Eraso-Pichot A.. (2014). The blood-brain barrier: structure, function and therapeutic approaches to cross it. Mol. Membr. Biol. 31, 152–167. 10.3109/09687688.2014.937468 25046533

[B36] Thiesen B. Jordan A. (2008). Clinical applications of magnetic nanoparticles for hyperthermia. Int. J. Hyperthermia. 24, 467–474. 10.1080/02656730802104757 18608593

[B37] Thorek D. L. Chen A. K. Czupryna J. Tsourkas A. (2006). Superparamagnetic iron oxide nanoparticle probes for molecular imaging. Ann. Biomed. Eng. 34, 23–38. 10.1007/s10439-005-9002-7 16496086

[B38] Van Landeghem F. K. H. Maier-Hauff K. Jordan A. Hoffmann K.-T. Gneveckow U. Scholz R.. (2009). Post-mortem studies in glioblastoma patients treated with thermotherapy using magnetic nanoparticles. Biomaterials 30, 52–57. 10.1016/j.biomaterials.2008.09.044 18848723

[B39] Varallyay P. Nesbit G. Muldoon L. L. Nixon R. R. Delashaw J. Cohen J. I.. (2002). Comparison of two superparamagnetic viral-sized iron oxide particles ferumoxides and ferumoxtran-10 with a gadolinium chelate in imaging intracranial tumors. Am. J. Neuroradiol. 23, 510–519. 11950637 PMC7975083

[B40] Wankhede M. Bouras A. Kaluzova M. Hadjipanayis C. G. (2012). Magnetic nanoparticles: an emerging technology for malignant brain tumor imaging and therapy. Exp. Rev. Clin. Pharmacol. 5, 173–186. 10.1586/ecp.12.1 22390560 PMC3461264

[B41] Wust P. Hildebrandt B. Sreenivasa G. Rau B. Gellermann J. Riess H.. (2002). Hyperthermia in combined treatment of cancer. Lancet Oncol. 3, 487–497. 10.1016/S1470-2045(02)00818-5 12147435

[B42] Yanase M. Shinkai M. Honda H. Wakabayashi T. Yoshida J. Kobayashi T. (1997). Intracellular hyperthermia for cancer using magnetite cationic liposomes: *ex vivo* study. Jpn. J. Cancer Res. 88, 630–632. 10.1111/j.1349-7006.1997.tb00429.x 9310134 PMC5921484

